# *REST* and *RASSF1A* Tumor Suppressor Genes in Peripheral Blood: Potential Molecular Markers in Breast Cancer

**DOI:** 10.3390/ijms27062752

**Published:** 2026-03-18

**Authors:** Maria Eduarda R. de Oliveira, Marina P. Silva, Estella F. Silvestri, Samia F. Sanches, Isabella D. R. Trufelli, Ludmila F. B. Fabbrini, Glaucia L. da Veiga, Fernando Luiz A. Fonseca, Beatriz da C. A. Alves

**Affiliations:** 1 Laboratório de Análises Clínicas, Centro Universitário FMABC, Av. Principe de Gales, 821, Santo André 09060-950, São Paulo, Brazil; maria.roliveira@aluno.fmabc.net (M.E.R.d.O.); marina.paschoal@aluno.fmabc.net (M.P.S.); estella.silvestri@aluno.fmabc.net (E.F.S.); samia.sanches@aluno.fmabc.net (S.F.S.); isabella.trufelli@fmabc.br (I.D.R.T.); ludmilla.fabbrini@fmabc.br (L.F.B.F.); grlveiga@gmail.com (G.L.d.V.); profferfonseca@gmail.com (F.L.A.F.); 2 Departamento de Ciências Farmacêuticas, Universidade Federal de São Paulo, R. Prof. Artur Riedel, 275, Diadema 09972-270, São Paulo, Brazil

**Keywords:** neoplasm, tumor suppressor gene, biomarker, gene expression, peripheral blood

## Abstract

Tumor suppressor genes, such as *RASSF1A* and *REST*, play a central role in regulating cell proliferation. *RASSF1A* is frequently inactivated in various cancers, being associated with poor prognosis and metastasis. REST loss promotes the activation of genes related to invasion and estrogen sensitivity. We aimed to evaluate the expression of *REST* and *RASSF1A* in peripheral blood of breast cancer patients at different treatment stages and to associate the results with clinical and laboratory variables. Peripheral blood samples from breast cancer patients were collected at diagnosis and at 3 and 6 months after the start of chemotherapy; blood samples from healthy women were also collected. Gene expression was quantified by qPCR and associated with clinical variables. *REST* expression was significantly lower in patients (*p* < 0.0001), showing a negative correlation with the BIRADS classification and an AUC of 0.72. *RASSF1A* showed no significant difference between groups but was negatively correlated with heparanase (r = −0.4213; *p* < 0.0001). No relevant variations in gene expression were observed among the serial collections, nor associations with histological type. Downregulation of *REST* expression in the peripheral blood of breast cancer patients suggests its potential as an auxiliary biomarker for diagnosis and risk stratification. *RASSF1A* was correlated with mechanisms associated with tumor progression but did not differentiate patients from controls.

## 1. Introduction

Breast cancer is the most frequently diagnosed neoplasm among women worldwide, accounting for approximately 10% of all cancer diagnoses [1]. Malignant neoplasms are essentially understood as disorders of cell growth, since cancer development is a multistep process characterized by uncontrolled cell proliferation, invasion, and metastasis resulting from the accumulation of genetic and epigenetic alterations [2]. In this context, tumor suppressor genes (TSGs) play a crucial role in regulating and suppressing cell proliferation [3].

TSGs are involved in DNA repair, cell division control, apoptosis induction, and metastasis suppression [4], and their loss of function can initiate and accelerate cancer progression [5]. p53 is the most extensively studied TSG, frequently mutated in several human cancers and associated with increased susceptibility to tumor development [6]. Another relevant suppressor gene is *RASSF1A* (Ras Association Domain Family Member 1A), located in the 3p21.3 region, which is known for its susceptibility to epigenetic alterations in various cancers [7]. The inactivation of *RASSF1A* is common in multiple cancer types and is associated with hypermethylation of its promoter region or loss of heterozygosity [7]. Studies indicate that up to 15% of tumors may contain inactivating point mutations in *RASSF1A* [8]. In bladder cancer, for example, lower *RASSF1A* expression is associated with reduced Disease-Free Survival (DFS), higher recurrence rates, and muscle invasion [9].

The *REST* gene (RE-1 Silencing Transcription Factor), also known as *NRSF* or *XBR*, is a transcriptional repressor involved in cellular processes such as differentiation, migration, apoptosis, and pluripotency [10]. Although initially considered neuron-specific, *REST* can repress neuronal genes in non-neuronal cells by binding to the neuron-restrictive silencer element (NRSE) present in these genes [11,12]. *REST* also acts in various non-neuronal tissues, including the heart, pancreas, skin, eyes, and blood vessels, and is implicated in several types of cancer [10]. In these contexts, it can function either as a tumor suppressor or as an oncogene, depending on the cellular microenvironment [13]. For example, in gliomas and medulloblastomas, increased *REST* expression is associated with tumor progression, suggesting an oncogenic role [14,15]; however, in cholangiocarcinoma, *REST* expression is reduced, while in hepatocellular carcinomas, there is no significant alteration compared to normal liver tissues [16]. In breast cancer, however, its function is lost in approximately 20% of tumors and is associated with more aggressive phenotypes and poorer prognosis [17,18]. This loss promotes tumor progression through estrogen sensitization and activation of genes such as MMP24 and CEMIP, which are linked to invasion and metastasis [18]. Moreover, *REST*-deficient breast tumors exhibit increased expression of target genes and a distinct phenotype [19]. The interaction of REST with factors such as LIN28A is also essential for the growth of these tumors, reinforcing its importance in breast cancer progression [20].

Several studies indicate that the analysis of genetic signatures in peripheral blood cells is a promising tool for identifying diseases [19,21]. Due to their easy accessibility, minimally invasive nature, and low cost, blood samples stand out as an attractive alternative for diagnostic applications [22]. In the case of breast cancer, the development of the cancer induces detectable changes in the gene expression profile of whole blood, reflecting the response of circulating blood cells to the presence of the tumor. This occurs because tumor–host interactions extend beyond the tumor microenvironment, as cancer depends on the exploitation of normal physiological processes by tumor cells [23]. In this context, Sharma et al. [24] identified a peripheral blood gene signature capable of distinguishing healthy women from those with breast cancer, with an accuracy of 79.5%, sensitivity of 80.6%, and specificity of 78.3%. Similarly, Dumeaux et al. [23] identified a 50-gene signature, mostly related to immune response, with 72.9% accuracy, 83.1% sensitivity, and 62.7% specificity. Furthermore, data from our group demonstrated that *HIF1α* (Hypoxia Inducible Factor 1 Subunit Alpha) gene expression in peripheral blood distinguishes women with and without breast cancer with an accuracy greater than 90% [25].

Although the involvement of *REST* and *RASSF1A* in breast cancer has already been reported, the expression profiles of these genes in the peripheral blood of patients with this neoplasm, as well as their diagnostic potential, have not yet been explored. Therefore, this study aimed to evaluate the expression of *REST* and *RASSF1A* in serial peripheral blood samples (collected at diagnosis and at 3 and 6 months after the initiation of chemotherapy) from women with breast cancer, comparing them to healthy women, to investigate the diagnostic and/or prognostic potential of these genes as biomarkers in liquid biopsy.

## 2. Results

In this study, samples from 120 breast cancer patients and 81 healthy women with recent BIRADS scores ≤ 3 (values indicating no suspected lesions) were included, with 6 classified as BIRADS 1 (7.4%), 71 as BIRADS 2 (87.7%), and 4 as BIRADS 3 (4.9%). The healthy women had a mean age of 54.1 years (±10.4) while the mean age of the patients was 52.6 years (±11.1). Considering treatment, the distribution of therapeutic regimens showed a predominance of cytotoxic chemotherapy, particularly anthracycline- and taxane-based regimens. Sequential chemotherapy with a taxane (doxorubicin plus cyclophosphamide followed by a taxane administered weekly or every three weeks) was the most frequent regimen, accounting for 78 (65%) cases. Anthracycline-based regimens (doxorubicin plus cyclophosphamide or 5-fluorouracil plus doxorubicin plus cyclophosphamide) were administered to 26 patients (21.7%). Classical polychemotherapy with cyclophosphamide, methotrexate, and 5-fluorouracil (CMF) was used in 6 cases (5%) whereas taxane monotherapy (paclitaxel or docetaxel) was observed in 3 patients (2.5%). Among endocrine therapies, 4 (3.3%) patients received tamoxifen, while 3 (2.5%) were treated with aromatase inhibitors (anastrozole or letrozole). Overall, there was a greater use of combined regimens containing anthracyclines and taxanes, reflecting contemporary clinical practice in the systemic treatment of breast cancer. The clinical characteristics of the patients included and their treatment regimens are described in Table 1.

**Figure 1:**
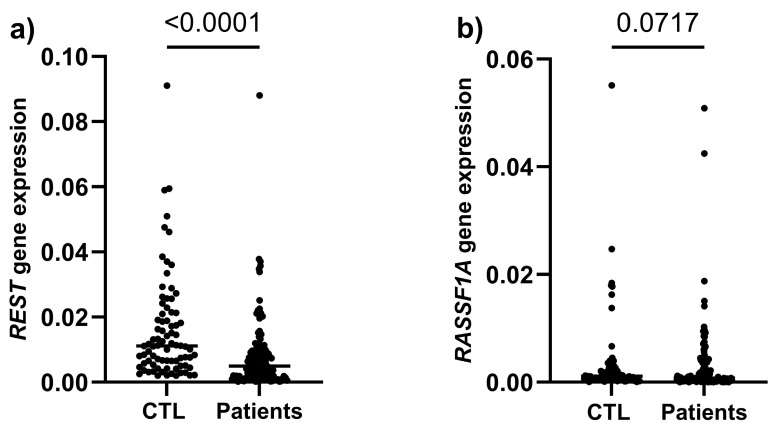
Graphical representation of *REST* (**a**) and *RASSF1A* (**b**) gene expression in healthy women (CTL) and patients.

The evaluation of the diagnostic power of the markers was performed by comparing the expression of the *REST* and *RASSF1A* genes between the control group (healthy women) and breast cancer patients. Breast cancer patients showed significantly reduced *REST* levels compared to the control group (*p* < 0.0001), whereas for *RASSF1A* the difference did not reach statistical significance (*p* = 0.0717) (Figure 1).

**Figure 2:**
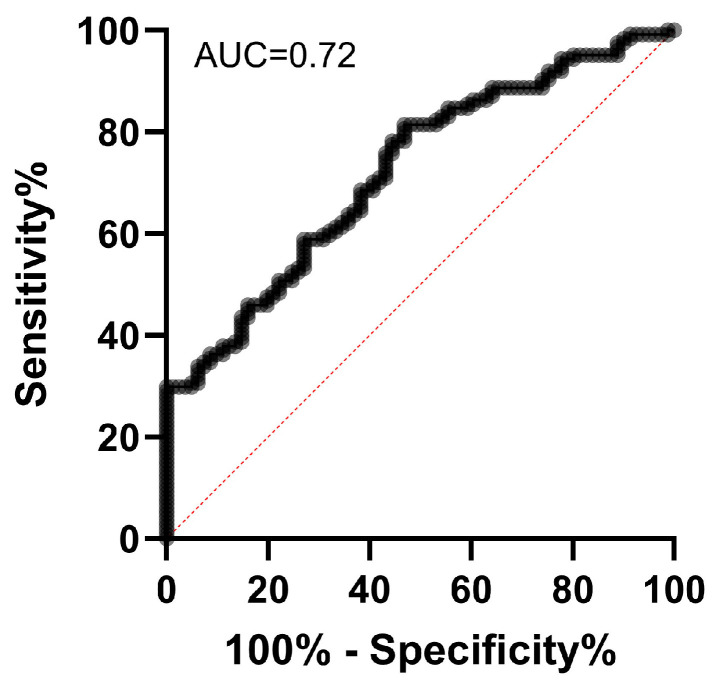
Graphical representation of the ROC curve and AUC generated for *REST* in breast cancer patients and healthy women.

Using the *REST* expression values from patients and the control group, a ROC (Receiver Operating Characteristic) curve was generated to determine the marker’s sensitivity and specificity, as well as to establish a cutoff value capable of distinguishing between these two populations. The ROC analysis for *REST* showed an area under the curve (AUC) of 0.72 (95% CI: 0.65–0.79; *p* < 0.0001) (Figure 2), indicating moderate performance in differentiating breast cancer patients from the control group. For this AUC, the sensitivity was 80.6%, specificity 53%, and the cutoff value was 0.01. These results suggest that *REST* has potential diagnostic utility, with accuracy above chance, although not high enough for use as a standalone test. The ROC curve for *RASSF1A* yielded insignificant results, as this marker was not effective in distinguishing diseased women from healthy ones.

The prognostic potential of the markers was assessed by monitoring gene expression throughout treatment. No significant changes in *REST* or *RASSF1A* expression were observed across the three time points (at diagnosis, and 3 and 6 months after treatment), indicating that their peripheral blood levels are not predictive of treatment response.

**Figure 3:**
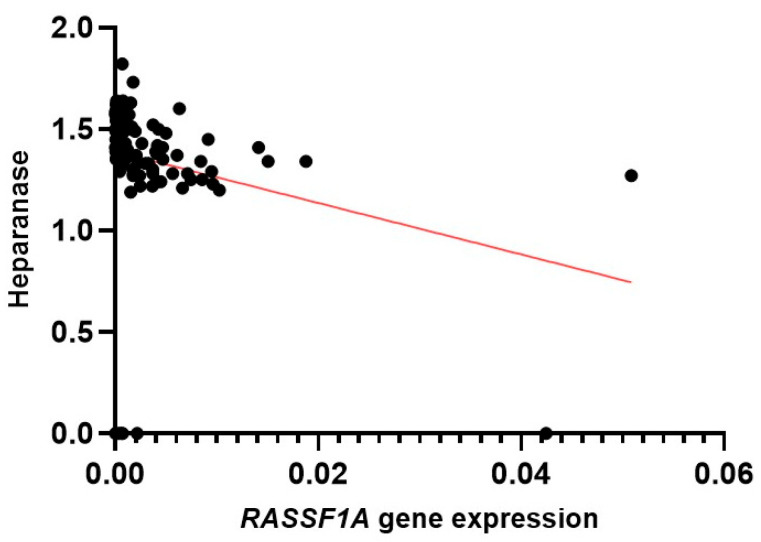
Correlation between *RASSF1A* gene expression and heparanase.

*REST* and *RASSF1A* expressions were also correlated with traditional tumor markers such as ER, PR, Her2, and heparanase. In this analysis, *REST* showed no significant association with any of the evaluated parameters. In contrast, *RASSF1A* exhibited a moderate negative correlation with heparanase (r = −0.4213; 95% CI: −0.5599 to −0.2597; *p* < 0.0001) (Figure 3), indicating that lower expression levels of this gene in peripheral blood are associated with higher levels of this enzyme, which is recognized for its role in extracellular matrix degradation and potential facilitation of metastatic processes.

When comparing *REST* and *RASSF1A* gene expression according to tumor histological type, no statistically significant differences were observed among the evaluated subtypes (Luminal, triple-negative, and HER2-enriched). These findings indicate that the expression of these genes in peripheral blood did not vary substantially according to tumor histology. Similarly, the different treatments received by the patients did not interfere with the expression of the genes under study, nor did the expression of these genes interfere with the response to treatment (*p* > 0.05).

## 3. Discussion

Breast cancer is the most frequent neoplasm in women worldwide, involving genetic and epigenetic alterations that affect tumor suppressor genes such as *RASSF1A* and *REST*. *RASSF1A* inactivation by hypermethylation and *REST* functional loss are associated with increased aggressiveness and metastatic potential. Although their roles in tumor tissue are established, little is known about their expression in peripheral blood. As liquid biopsy is a non-invasive alternative for diagnosis and monitoring, this study aimed to investigate these genes in blood and assess their potential as biomarkers in breast cancer.

Our results suggest that breast cancer patients exhibit a significant decrease in *REST* expression in peripheral blood compared to healthy women. Whole peripheral blood was used, which may contain not only blood cells but also circulating tumor cells (CTCs) and cell-free RNA (cfRNA). *REST* expression in leukocytes may influence inflammatory responses, affecting cytokine production and interactions between platelets and leukocytes, including markers such as IL-6 [26,27]. REST also regulates leukocyte adhesion molecules (ICAM-1, VCAM-1) and cell recruitment during inflammation [28], and post-transcriptional modifications, such as ubiquitination, can alter its nuclear localization and repressor function [29]. Reduced REST levels in leukocytes may contribute to autoimmune and inflammatory diseases [30]. Chronic inflammation is associated with cancer predisposition, and the tumor microenvironment is influenced by inflammatory cells. Macrophages and neutrophils can promote tumor growth through cytokine and growth factor secretion [31]. M2 macrophages are linked to adverse outcomes due to angiogenesis and extracellular matrix degradation, facilitating metastasis [31,32]. The decreased REST observed aligns with studies showing loss of function in ~20% of breast tumors, associated with more aggressive phenotypes and poor prognosis [17,33]. The ROC curve for *REST* (AUC = 0.7223) indicates moderate performance in distinguishing patients from controls, consistent with literature on blood biomarkers in breast cancer, which rarely achieve high accuracy individually but can be informative in combined panels [23,24].

*RASSF1A*, by its turn, is a well-established tumor suppressor gene typically inactivated in cancer through promoter hypermethylation rather than consistent reductions in mRNA expression [7,34,35,36,37,38]. In the present study, only transcript levels were assessed by qPCR in peripheral blood samples without evaluating epigenetic alterations; therefore, the lack of a statistically significant reduction in *RASSF1A* levels among patients does not exclude the possibility of functional inactivation mediated by DNA methylation or other regulatory mechanisms. This absence of significance may also relate to biological variability, sample size, or dependence on specific disease stages and tumor molecular profiles. Notably, no association was observed between *RASSF1A* expression and different tumor subtypes or classical tumor markers, except for a moderate negative correlation with heparanase. This finding is relevant considering heparanase is normally expressed in placental tissue, immune system cells, and platelets [39,40], and primarily functions to cleave heparin sulfate, an essential extracellular matrix component. This degradation promotes matrix remodeling, releasing bound cytokines and growth factors, thereby facilitating immune cell migration, angiogenesis, inflammation, tissue repair, and metastatic dissemination [41,42]. Beyond enzymatic activity, heparanase regulates gene expression, stimulates cell adhesion, and exhibits pro-coagulant activity. In the oncological context, its overexpression enhances neoplastic cell growth and dissemination, resulting in poorer clinical outcomes [43,44,45]. Gene expression deregulation drives pathological heparanase upregulation in the tumor microenvironment, promoting abnormal extracellular matrix remodeling and releasing heparin sulfate-bound proteins with pro-tumor potential [46]. Thus, lower *RASSF1A* levels accompanied by higher heparanase expression may indicate a microenvironment more conducive to cancer progression. Future studies integrating methylation analysis and expression profiling may provide a more comprehensive understanding of *RASSF1A* regulation in liquid biopsy samples.

No significant differences in *REST* or *RASSF1A* expression were observed across the serial collections (diagnosis, 3 and 6 months) or among different histological subtypes. Also, we did not identify an association between the expression of *REST* and *RASSF1A* and response to chemotherapy regimen. Similarly, the treatment did not induce detectable changes in the expression of these genes. These results indicate that, in the context investigated, *REST* and *RASSF1A* do not directly contribute to determining tumor sensitivity nor participate in chemotherapy-induced transcriptional responses—a finding that contrasts with the behavior of TSGs classically implicated in therapeutic response. For instance, alterations in TP53 influence the capacity to induce apoptosis following DNA damage, thereby impacting the efficacy of cytotoxic agents [47]. Similarly, BRCA1 deficiency is associated with increased sensitivity to drugs that induce double-strand breaks [48], whereas PTEN loss may modulate cellular survival pathways related to therapeutic resistance [49]. Additionally, genes such as *CDKN1A* may exhibit induced expression following exposure to chemotherapeutic agents, reflecting activation of genomic damage response mechanisms [50]. This suggests that, within the analyzed time frame, the expression of these genes was not substantially modulated by treatment and did not vary between tumor subtypes, although longitudinal studies with longer follow-up and larger sample sizes are needed to clarify this issue. Overall, the results indicate that *REST* has greater potential as a liquid biopsy biomarker for breast cancer than *RASSF1A*, particularly as an auxiliary diagnostic marker and possibly as an indicator of tumor aggressiveness. Nonetheless, the clinical use of *REST* alone is limited, and its greatest applicability is likely in combination with other genes and serum markers, forming molecular signatures with higher accuracy.

The reduction in *REST* expression observed in peripheral blood samples suggests that this gene may have potential as an exploratory biomarker in breast cancer. However, given the moderate diagnostic performance observed (AUC = 0.72) and the absence of direct comparison with tissue biopsy or established diagnostic modalities, these findings should be interpreted with caution. At this stage, *REST* cannot be considered a replacement for conventional diagnostic approaches, nor does our study demonstrate improved diagnostic precision over current standards. Instead, our results indicate that *REST* expression in peripheral blood may contribute to future multi-marker panels, pending validation in larger and independent cohorts.

Liquid biopsy approaches have been increasingly investigated in oncology due to their minimally invasive nature and potential for longitudinal monitoring in cancer diagnosis, monitoring, and prognosis [51,52]. However, the clinical utility of individual biomarkers requires robust validation, including assessment of incremental diagnostic value over established methods.

Our results demonstrated that *REST* gene expression is significantly reduced in the peripheral blood of breast cancer patients compared to healthy women, showing a moderate performance in distinguishing groups according to ROC curve analysis. These finding reinforce the potential of *REST* as an auxiliary biomarker for diagnosis in this neoplasm. Although *RASSF1A* is recognized as an important tumor suppressor gene, it did not show a significant reduction in patients but exhibited a negative correlation with heparanase, which may indicate an indirect role in tumor invasion processes. The lack of significant variation in the expression of both genes across serial samples, among histological subtypes and across different treatment regimens suggests that their detection in liquid biopsy does not reflect short-term dynamic changes, specific histopathological features, or response to therapy.

This study presents several limitations. Although the sample size detected significant *REST* expression differences, it may limit generalizability and subgroup analysis power. Selection bias is possible due to single-center recruitment and control composition, while the lack of an independent validation cohort necessitates external validation. Methodologically, only mRNA expression was evaluated; *RASSF1A* is frequently regulated by promoter hypermethylation, and epigenetic alterations were not assessed in this study. Thus, relevant regulatory mechanisms may not have been captured. Finally, analyzing whole blood leaves unclear whether alterations reflect tumor-derived material or a systemic host response, warranting further mechanistic investigation.

Future studies with larger sample sizes and integration with other biomarkers may further clarify the roles of *REST* and *RASSF1A* as diagnostic and prognostic tools in breast cancer, supporting their use in personalized medicine strategies.

## 4. Materials and Methods

### 4.1. Patients

To evaluate the expression of *RASSF1A* and *REST* and their association with pathological variables such as stage, progression, and hormonal markers (Her2, PR, and ER), samples from breast cancer patients were obtained from the Oncology Service of the Centro Universitário FMABC. Peripheral blood samples were collected from these patients at diagnosis and at 3 and 6 months after the initiation of chemotherapy. As controls, peripheral blood samples were obtained from women known to be free of neoplasia (women with recent clinical, laboratory, or imaging examinations ruling out breast alterations), matched by age. Peripheral blood samples were collected by venipuncture into PAXgene Blood RNA Tubes (PreAnalytiX, Hombrechtikon, Switzerland, cat. no. 762165), which contain RNA stabilizers. This study was approved by the Research Ethics Committee of the Centro Universitário FMABC (approval no. 346.712, 15 July 2024), and all participants signed the informed consent form.

### 4.2. RNA Isolation and cDNA Synthesis

Total RNA was extracted from peripheral blood samples using the PAXgene Blood RNA Kit (PreAnalytiX^®^, cat. no. 762164) according to the manufacturer’s instructions and quantified with a Qubit 4 fluorometer (ThermoFisher Scientific, Waltham, MA, USA, cat. no. Q33238) using the Qubit RNA BR Assay Kit (ThermoFisher Scientific, cat. no. Q10211). For cDNA synthesis, 500 ng of total RNA and the QuantiTect Reverse Transcription Kit (Qiagen, Hilden, Germany, cat. no. 205311) were used.

### 4.3. qPCR

The expression of *RASSF1A* and *REST* genes was evaluated by real-time PCR (qPCR). Specific primers for each selected gene were designed using the Primer3 Input 0.4.0 software, available at https://primer3.ut.ee/ (accessed on 20 February 2025). The designed primer sequences were checked for specificity using the Primer-BLAST tool, available at https://www.ncbi.nlm.nih.gov/tools/primer-blast/ (accessed on 20 February 2025). The sequences of the designed primers cannot be disclosed due to patent protection concerns. Both primer pair hybridizes to a conserved region of the target genes mRNA. For normalization of the relative expression of the target genes, the mean expression values of the *RPL13a* gene—which encodes a ribosomal protein component of the 60S subunit—were used. Real-time PCR amplification reactions were performed on Applied Biosystems 7500 Real-Time PCR System (Applied Biosystems, Foster City, CA, USA) in a final volume of 15 µL containing: 1X SYBR Green mix (QuantiTect SYBR Green PCR Kit, Qiagen, cat. no. 204054), 15 pmol of each specific primer, and 1.5 µL of cDNA. The cycling parameters consisted of an initial hot-start step at 95 °C for 10 min, followed by 40 cycles of 95 °C for 15 s and 60 °C for 25 s. Gene expression was determined using the 2^^(−ΔCq)^ formula.

### 4.4. Statistical Analysis

Initially, a descriptive analysis was performed in which categorical variables were expressed as absolute and relative frequencies, and quantitative variables were summarized as means, standard deviations, minimum, and maximum values. To evaluate the expression of the proposed genes in breast cancer patients and healthy women, Student’s *t*-test was applied. In addition, repeated measures analysis of variance (ANOVA) was used to compare gene expression evolution over time and according to covariates such as disease stage, progression, time to progression, age, and the presence of estrogen and progesterone receptors. Statistical analyses were performed using the NCSS software package (version 24.0.x). 

## Figures and Tables

**Table 1:** Patients’ clinical characteristics.

Clinical Characteristics	N	%
**Clinical Stage**		
0/I	32	26.6
II	59	49.2
III	29	24.2
**Progression**		
Negative	110	91.6
Positive	10	8.4
**Estrogen receptor**		
Negative	30	25.0
Positive	90	75.0
**Progesterone receptor**		
Negative	50	41.6
Positive	70	58.4
**Her_ihc**		
Negative	28	23.4
+/3+	38	31.6
++/3+	24	20.0
+++/3+	30	25.0
**Immunohistochemical tumor subtype**		
Luminal (HR+)	90	75.2
Triple-negative (HR−/HER2−)	9	7.4
Her2-enriched (HR−/HER2+)	21	17.4
**Treatment Regimen**		
Polychemotherapy	6	5.0
Anthracycline-based chemotherapy	26	21.7
Sequential taxane-based chemotherapy	78	65.0
Taxane monotherapy	3	2.5
Hormone therapy	4	3.3
Aromatase inhibitor	3	2.5
	**Median (p.25; p.75)**	**Min.; Max.**
Age	53 (47.5; 61.5)	27; 78

+/3+, ++/3+, or +++/3+ represent the standard positivity index. p.25; p.75: 25–75; Min., Minimum; Max., Maximum.

## Data Availability

The raw data supporting the conclusions of this article will be made available by the authors on request.

## Abbreviations

The following abbreviations are used in this manuscript:

AUC	Area Under the Curve
ANOVA	Analysis of Variance
BI-RADS	Breast Imaging Reporting and Data System
cDNA	Complementary DNA
cfRNA	Cell-Free RNA
CI	Confidence Interval
CTC	Circulating Tumor Cell
ctDNA	Circulating Tumor DNA
ctRNA	Circulating Tumor RNA
DFS	Disease-Free Survival
DNA	Deoxyribonucleic Acid
ER	Estrogen Receptor
HER2	Human Epidermal Growth Factor Receptor 2
HIF1α	Hypoxia-Inducible Factor 1 Alpha
HR	Hormone Receptor
ICAM-1	Intercellular Adhesion Molecule 1
IL-6	Interleukin 6
LIN28A	Lin-28 Homolog
NRSE	Neuron-Restrictive Silencer Element
NRSF	Neuron-Restrictive Silencer Factor
PCR	Polymerase Chain Reaction
PR	Progesterone Receptor
qPCR	Quantitative Polymerase Chain Reaction
RASSF1A	Ras Association Domain Family Member 1A
REST	RE-1 Silencing Transcription Factor
ROC	Receiver Operating Characteristic
RNA	Ribonucleic Acid
TSG	Tumor Suppressor Gene
